# Oxidative stress-dependent activation of collagen synthesis is induced in human pulmonary smooth muscle cells by sera from patients with scleroderma-associated pulmonary hypertension

**DOI:** 10.1186/s13023-014-0123-7

**Published:** 2014-08-01

**Authors:** Francesco Boin, Gian Luca Erre, Anna Maria Posadino, Annalisa Cossu, Roberta Giordo, Gaia Spinetti, Giuseppe Passiu, Costanza Emanueli, Gianfranco Pintus

**Affiliations:** 1 Johns Hopkins Scleroderma Center, Division of Rheumatology, Johns Hopkins University, 5200 Eastern Avenue, Baltimore, MD, USA; 2 Department of Clinical and Experimental Medicine, Rheumatology Unit, University of Sassari, Viale San Pietro 8, Sassari, Italy; 3 Laboratory of Vascular Biology, Department of Biomedical Sciences, University of Sassari, Viale San Pietro 43/B, 07100, Sassari, Italy; 4 Laboratory of Diabetological Research, IRCCS MultiMedica, Milan, Italy; 5 Bristol Heart Institute, University of Bristol, Bristol, England, UK

**Keywords:** Systemic sclerosis, Pulmonary arterial hypertension, Oxidative stress, Vascular smooth muscle cells

## Abstract

Pulmonary arterial hypertension is a major complication of systemic sclerosis. Although oxidative stress, intima hyperplasia and a progressive vessel occlusion appear to be clearly involved, the fine molecular mechanisms underpinning the onset and progression of systemic sclerosis-associated pulmonary arterial hypertension remain largely unknown. Here we shows for the first time that an increase of NADPH-derived reactive oxygen species production induced by sera from systemic sclerosis patients with pulmonary arterial hypertension drives collagen type I promoter activity in primary human pulmonary artery smooth muscle cells, suggesting that antioxidant-based therapies should be considered in the treatment of systemic sclerosis-associated vascular diseases.

## Findings

### Background

Pulmonary arterial hypertension (PAH) is a serious complication associated with significant morbidity and unfavorable outcomes in systemic sclerosis (SSc) [1]. This disease process is characterized by proliferative and fibrotic events involving all three layers of the blood vessel wall in the pulmonary circulation resulting from impaired function of endothelial and vascular smooth muscle cells (VSMCs) [2],[3]. Under homeostatic conditions, VSMCs exhibit a contractile phenotype, which regulate blood vessel diameter by virtue of stretch-sensing mechanisms. During vascular injury, a “phenotypic switch” can occur in response to pro-inflammatory, hypoxic and mitogenic stimuli, conferring to VSMCs the ability to migrate into intimal layers, differentiate, proliferate and synthesize extracellular matrix [4]. Inadequate regulation of this process contributes to the progressive narrowing and obliteration of pulmonary arterioles in SSc-PAH. Oxidative stress has been previously linked to vascular dysfunction, intimal hyperplasia and progressive vessel occlusion in SSc [5]. It is possible that circulating pro-oxidant factors may be involved in the pathogenesis of SSc-PAH through their ability to induce VSMCs activation and phenotypic switch. In order to verify this hypothesis, we investigated the production of reactive oxygen species (ROS) and collagen synthesis in primary human pulmonary artery smooth muscle cells (HPASMCs) exposed to serum obtained from SSc patients with or without PAH confirmed by right heart catheterization (RHC) and healthy donors (HD) (Table 1).

**Table 1:** Patient demographics and clinical characteristics

**Variables**	**No PAH (N = 17)**	**PAH (N = 19)**	**HD (N = 14)**	**p value**^**§**^
Age at serum sampling (years)*	53.3 ± 11.6	64.0 ± 9.4	54.1 ± 10.4	0.009
Female	15 (88)	16 (84)	15 (85)	0.727
Race				
White	14 (82)	16 (84)	12 (80)	0.881
Black	3 (18)	3 (16)	3 (20)	
Smoking status				
Never	9 (53)	10 (53)	8 (53)	0.280
Past	6 (35)	9 (47)	5 (33)	
Current	2 (12)	0	2 (13)	
SSc types				
Limited	11 (65)	16 (84)		0.177
Diffuse	6 (35)	3 (16)		
mRSS* (range 0–51)	5.5 ± 6.1	7.3 ± 10.3		0.567
SSc duration (RP onset)*, years	14.0 ± 12.6	21.7 ± 9.4		0.008
SSc duration (1st non-RP symptom)*, years	10.5 ± 7.3	18.5 ± 9.5		0.010
RP severity score* (range 0–4)	1.6 ± 0.8	2.0 ± 1.0		0.194
Heart severity score* (range 0–4)	0.2 ± 0.7	1.2 ± 1.7		0.062
Lung severity score* (range 0–4)	1.1 ± 1.3	3.1 ± 1.3		<0.001
Hemodynamics (RHC)				
mPAP* (mm Hg)	NA	35.2 ± 8.1		NA
PCWP* (mm Hg)	NA	11.5 ± 4.0		NA
FVC* (% predicted)	81.9 ± 22.9	73.1 ± 9.9		0.149
DLCO* (% predicted)	78.2 ± 23.0	48.7 ± 16.8		<0.001
RLD^**†**^	6 (35)	6 (32)		0.813
eRVSP*	24.0 ± 6.3	65.2 ± 19.9		<0.001
Autoantibody status				
ACA	4 (24)	10 (53)		0.07
Anti-Scl-70	7 (41)	1 (5)		0.01
Anti-RNA-polymerase 3	2 (12)	0		0.124
Medication use (current)				
Immunosuppressants^‡^	5 (29)	5 (26)		0.836
Calcium channel blocker	10 (59)	7 (37)		0.187
Endothelin receptor antagonist	1 (6)	6 (32)		0.052
Phosphodiesterase 5 inhibitor	4 (24)	11 (58)		0.037
Prostanoid	0	0		NA
Statin	6 (35)	5 (26)		0.559
Aspirin	5 (29)	5 (26)		0.836

All values are given as number (%) unless otherwise specified.

*Mean ± SD. ^**†**^The presence of RLD was defined by a FVC < 70% of predicted. ^‡^Use of immunosuppressants include cyclophosphamide, mycophenolate, methotrexate, hydroxycholorquine or prednisone. ^§^*P* values were determined by Fisher’s exact test or the Wilcoxon rank-sum test, as appropriate.

ACA, anticentromere antibody; DLCO, diffusion capacity of lung for carbon monoxide; eRVSP, estimated right ventricular systolic pressure by echocardiography; FVC, forced vital capacity; HD, healthy donors; mRSS, modified Rodnan skin score; mPAP, mean pulmonary artery pressure; PAH, pulmonary arterial hypertension, PCWP, pulmonary capillary wedge pressure, RHC, right heart catheterization, RLD, restrictive lung disease; RP, Raynaud’s phenomenon; Scl-70, topoisomerase I; SSc, systemic sclerosis.

### Methods

All SSc patients met the American College of Rheumatology criteria or had 3 of 5 features of the CREST (Calcinosis, Raynaud’s syndrome; Esophageal dysmotility; Sclerodactyly; Telangiectasia) syndrome [6]. SSc subjects were indicated as “No PAH” (n = 17) if their right ventricular systolic pressure (RVSP) estimated by echocardiogram was ≤35 mm Hg, while they were defined as “PAH” (n = 19) if the RVSP was >35 mm Hg and they underwent RHC showing a mean pulmonary artery pressure (mPAP) ≥ 25 mm Hg and pulmonary capillary wedge pressure (PCWP) ≤ 15 mm Hg. HD (n = 14) were selected through a screening questionnaire to rule out any underlying autoimmune or vascular disease. Intracellular ROS levels were assessed in HPASMCs using the oxidative stress indicator dichlorodihydrofluorescein-diacetate (H_2_-DCFDA), while collagen type-I (COL1A1) synthesis was investigated employing COL1A1-LV-tGFP, a GFP-based lentiviral vector (LV) driven by the human COL1A1 gene promoter [7],[8]. A red fluorescence protein-based LV (EF1α-LV-FP602) was used to normalize the cell transduction efficiency. In selected experiments, HPASMCs were pretreated before serum exposure with a NADPH oxidase specific inhibitor (NOX2ds-tat, formerly gp91ds-tat) [9]. SSc disease duration was calculated at the time of serum sampling from the onset of RP or from the first non-RP symptom. RP, heart and lung severity scores are reported as previously defined by Medsger et al. [10]. Study subjects were enrolled according to the protocol approved by the IRB after signing the consent form. Healthy donors were recruited through posted flyers and enrolled after passing a screening questionnaire aimed at excluding the presence of any underlying vascular or autoimmune disease.

### Results

**Figure 1:**
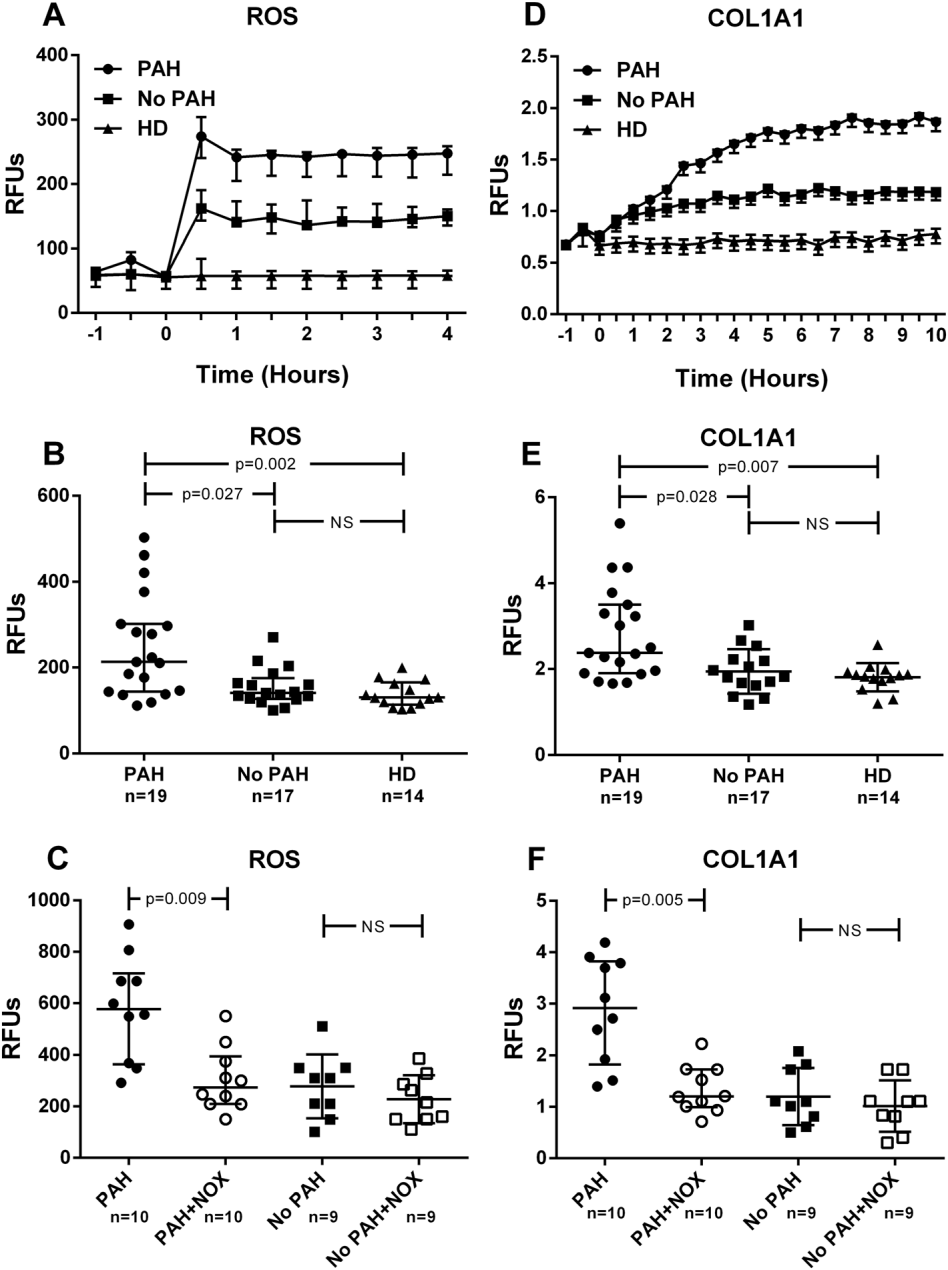
**Effect of sera on intracellular ROS levels and collagen promoter activity. (A-B)** Effects of SSc sera on human pulmonary artery smooth muscle cells (HPASMCs) intracellular ROS production. Before stimulation, sub-confluent HPASMCs were loaded with 10 μM of H2-DCFDA and then cultured in basal medium containing 10% (V/V) of sera from scleroderma (SSc) patients with pulmonary arterial hypertension (PAH), without PAH (No PAH) and healthy donors (HD) ^††^. Variations in intracellular ROS levels were kinetically determined in a 4 hour time-course experiment (Figure 1A) and values at 2 hours (steady state) used for comparison (Figure 1B). Fluorescence data were normalized for protein content and expressed as Relative Fluorescence Units (RFU). **(D-E)** Effects of SSc sera on HPASMCs collagen (COL1A1) promoter activation. Sub-confluent HPASMCs were transduced with lentiviral particles obtained from the COL1A1-LV-tGFP and EF1α-LV-FP602 lentivectors, and then cultured in basal medium containing 10% (V/V) of sera from PAH, no PAH and HD subjects. Variations of COL1A1 promoter activation were kinetically followed for 10 hours (Figure 1D) and values at 8 hours (steady state) used for comparison (Figure 1E). Data are normalized for transduction efficiency by reporting the ratio of COL1A1-LV-tGFP to EF1α-LV-FP602 Relative Fluorescence Units (RFU). **(C-F)** In selected experiments, HPASMCs were incubated for 1 hour with 5 μM NADPH oxidase specific inhibitor NOX2ds-tat (NOX) before treatment with SSc sera. ^††^Healthy donors were matched for gender, race and smoking status. Horizontal lines indicate the median with interquartile range. Kruskall–Wallis one-way analysis of variance followed by post-hoc Dunn’s test for multiple comparisons were used to detect differences among studied groups in Figures 1B and E. Wilcoxon matched-pairs signed rank test was used to determine meaningful differences between pre- and post-NOX treatment pairs in Figures C and F. All statistical analysis were performed using GraphPad Prism version 6.00 for Windows (GraphPad Software, San Diego, CA) and p-values <0.05 were considered to be statistically significant.

SSc patients enrolled in the study were mainly middle age, white women. Subjects with PAH were slightly older (64.0 ± 9.4 vs 53.3 ± 11.6; p = 0.009) and had longer disease duration (18.5 ± 9.5 vs 10.5 ± 7.3 years; p = 0.01). As expected, they exhibited higher lung severity scores (3.1 ± 1.3 vs 1.1 ± 1.3; p < 0.001) and a significantly lower diffusion capacity of lung for carbon monoxide (DLCO) (48.7 ± 16.8 vs 78.2 ± 23.0; p < 0.001) with comparable forced vital capacity (FVC), indicative of the underlying pulmonary vascular disease. The use of vasodilators (i.e. endothelin receptor antagonists and phosphodiesterase 5 inhibitors) was significantly higher in PAH patients. Intracellular ROS levels were kinetically determined in a 4-hour time-course (Figure 1A) and values at 2 hours (steady state) were used for comparison (Figure 1B). Sera from SSc-PAH patients significantly increased intracellular ROS levels in HPASMCs with median (interquartile range) of 213 (158) compared to subjects without PAH [141 (48); p = 0.027] and HD [130 (52); p = 0.002]. NOX2ds-tat effectively reduced induction of ROS by PAH-SSc sera (p = 0.009), implicating NADPH oxidase in this process (Figure 1C). Exposure of HPASMCs to SSc-PAH sera also resulted in progressive time-related increase of the COL1A1 promoter activity (Figure 1D) with values at 8 hours (steady state) significantly higher in cells exposed to PAH [2.375 (1.597)] compared to no-PAH [1.825 (0.612); p = 0.028] and HD [1.844 (0.265); p = 0.007] sera (Figure 1E). Similarly to ROS production, also this effect was inhibited by NOX2ds-tat pretreatment (p = 0.005) (Figure 1F), suggesting that phenotypic switch and collagen synthesis activation in HPASMCs may be driven by SSc-related PAH sera through NADPH-oxidase dependent ROS generation.

### Conclusion

This pilot study provides new evidence supporting the possibility that vascular disease and in particular PAH may be driven or maintained in SSc patients by pro-oxidant circulating factors acting, at least in part, through the activation of collagen synthesis in VSMCs. While more studies are needed to identify with precision these mediators and to define the molecular basis of their effect, our data provide further rationale for considering anti-oxidant therapies in the treatment or prevention of SSc-related pulmonary vascular disease.

## Abbreviations

PAH: Pulmonary arterial hypertension

SSc: Systemic sclerosis

VSMCs: Vascular smooth muscle cells

ROS: Reactive oxygen species

HPASMCs: Human pulmonary artery smooth muscle cells

RHC: Right heart catheterization

HD: Healthy donors

RVSP: Right ventricular systolic pressure

H_2_-DCFDA: Dichlorodihydrofluorescein-diacetate

COL1A1: Collagen type-I

LV: Lentiviral vector

FVC: Forced vital capacity

## Competing interest

Authors declare that they have no competing financial, professional or personal interests that might have influenced the performance or presentation of the described work.

## Authors’ contributions

All authors were involved in drafting the article or revising it critically for important intellectual content and all authors approved the final version. Study conception and design: FB, GLE, GP. Acquisition of data: FB, AMP, AC, RG, GP. Analysis and interpretation of data: FB, GLE, AMP, AC, RG, GS, GP, CE, GP.

## Authors’ information

CE is a British Heart Foundation Senior Research Fellow and a senior investigator of the Bristol National Institute of Health Research Biomedical Research Unit in Cardiovascular Disease. RG and AC are research fellow supported respectively by the Sardinia Region P.O.R. SARDEGNA, F.S.E. 2007-2013-Human Capital Objective-Line of Activity 1.3.1 and Bank of Sardinia Foundation.
